# Prevalence, response and associated factors of needlestick injury among health care workers in Orotta National Referral Hospital, Eritrea

**DOI:** 10.1186/s12913-024-11255-x

**Published:** 2024-07-27

**Authors:** Feven Beletse Negash, Amanuel Hailemichael, Enabi Haileslassie, Eyob Hawaz, Samuel Zerai, Zerabruk Tesfamariam, Laban Lebahati

**Affiliations:** 1 Department of Public Health, Asmara College of Health Sciences, Asmara, Eritrea; 2 Community Medicine and Primary Health Care, Orotta College of Medicine and Health Sciences, Asmara, Eritrea

**Keywords:** Needlestick injury, Health care workers, Orotta National Referral Hospital

## Abstract

**Background:**

Healthcare workers are at risk of occupational exposure to blood and other body fluids after sustaining needlestick injury which constitutes a risk for transmission of blood-borne pathogens such as Hepatitis B virus, Hepatitis C virus or Human Immune-deficiency Virus.

**Objectives:**

To assess the prevalence, response, and associated factors of needlestick injury by medical sharps among healthcare workers in Orotta National Referral Hospital, Asmara, Eritrea.

**Methods:**

Cross sectional study was conducted between September and December 2017 among healthcare workers. This was a census study whereby a total of 383 healthcare workers who had contact with sharp medical equipment were taken as study population. An aided self-administered questionnaire, checklist and key informant interviews were used as data collection tools. Analysis was done using Statistical Package for Social Sciences, version 22. Bivariate and binary logistic regression analyses were carried out and the level of significance was set at *P* < .05.

**Results:**

The prevalence of needlestick injury 12 months preceding the study was 37.1% (134/361). Midwives had the highest occurrence (45%) among others while adult intensive care unit were found to have higher prevalence of needlestick injury (61.5%) as compared to the other sections. As an immediate response to needlestick injury, only 15.7% washed the injured part with soap and water. The factors associated with needlestick injury include age > 40 years (AOR = .314, *p* = .05), marital status (married (AOR = 0.595, *p* = .05)), additional duty that made healthcare workers rush during working hours (AOR = 2.134, *p* = .002) and back bone problem (AOR = 2.239, *p* = .002).

**Conclusion:**

The overall finding of the study indicated that there was a great risk of contracting blood-borne infections among the healthcare workers especially midwives. Therefore, there is need for adequate supply of safety engineered devices, Hepatitis B vaccine, better reporting, and surveillance of needlestick injury cases at the hospital. Moreover, further research on assessment of the knowledge, attitude, and practice of healthcare workers toward occupational safety and health, particularly needlestick injury, is necessary.

**Supplementary Information:**

The online version contains supplementary material available at 10.1186/s12913-024-11255-x.

## Introduction

Needlestick injury (NSI), also referred as percutaneous injury, is the penetration of skin by needle or other sharp object, which is contaminated with blood, tissue, or other body fluid before the exposure [1]. NSIs are common accidents in the healthcare environment and are harmful as they facilitate the transmission of blood-borne diseases.

Despite the fact of the negative health effects NSIs cause, it is estimated that half of all occupational NSIs are not reported [2]. In 2007, the World Health Organization (WHO) estimated annual global NSIs at 2 million per year and another investigation estimated 3.5 million injuries yearly [3]. In China, about one million accidental NSIs have been reported every year, in which one healthcare worker (HCW) was exposed to NSI every 30 s [4]. It is apparent that each needle stick has the potential to be infectious but the estimates given are only the reported cases and about 40–70% cases of NSI are unreported in developing countries [5].

Among the more that 20 infectious diseases that can be transmitted by NSI, the most serious are Hepatitis C virus (HCV), Hepatitis B virus (HBV) and HIV. WHO estimated that in 2003 approximately 16,000 HCV infections, 66,000 HBV infections, and 1,000 HIV infections occur every year worldwide in HCWs from NSIs [6, 7]. For HCWs worldwide, the attributable proportions for percutaneous occupational exposure to HBV, HCV and HIV are 37%, 39% and 4.4%, respectively. In developing countries, 40–60% of HBV infection among HCWs was attributed professional hazard while in developed countries the attributed fraction was less than 10% due to vaccination coverage [8]. Although exposure to blood-borne pathogens is one of the dreadful hazards that HCWs face daily, it is also easily preventable. Over 80% of NSIs can be prevented with the use of safe needle devices and in conjunction with worker education and work practices injuries can be reduce by over 90% [9].

Fatigue, high workload, high pressure, or ignoring the risk can all increase the chances of NSIs [10]. NSIs are more common during night shifts and for less experienced people [2]. Low risk perception can be caused by poor knowledge about risk, or incorrect estimate of a particular patient’s risk [6]. In less developed countries, the risk of occupational transmission due to blood-borne pathogens is increased due to excessive handling of contaminated needles [11]. Neither the prevalence of needlestick injury nor the factors associated with it have been well understood among Sub-Saharan Africa [12]. Such factors are irregular utilization of protective gear, type of occupation of HCWs, disposing of contaminated needle, recapping of needles and drawing of blood [13]. Moreover, healthcare workers who followed universal precautions were 66% less likely to have NSI than those who did not adhere to recommendations [14].

Up to 90% of injuries due to NSIs occur in developing nations, however, studies showed that reporting of NSIs are less as compared to developed nations [15]. In Eritrea, very little is known about the prevalence of NSI. The unpublished studies in two tertiary hospitals in Eritrea that the authors could find, showed that 71% (of 76 respondents) and 75% (of 60 respondents) sustained NSI during the study period of December to January 2011 and March to May 2013, respectively. However, contributing factors were not addressed in those studies.

The objective of this study is to address NSI and aims at assessing its occurrence among HCW and the various associated factors.

## Methods

### Study design and setting

This is a cross sectional study conducted in 2017 from 1^st^ September to 31^st^ December at Orotta National Referral Hospital (ONRH) in Asmara, Eritrea, which is a tertiary public and teaching hospital.

### Study population

The study population was HCWs who were in direct contact with sharp medical equipment and who were likely to be exposed to NSI. Health care in the hospital is provided by 712 HCWs (Statistical Administrative Record of ONRH, 2017) out of which 383 were the study population. This was a census study where HCWs that worked at least one year in the study area, preceding the study and who had contact with needles were included.

### Data collection techniques

An anonymous self-administered questionnaire was developed after review of relevant literature and adopted to the local situation it is attached with the manuscript as supplementary file.

Pilot study was conducted on a random sample of 30 health care workers in Halibet Regional Referral Hospital in Asmara, Eritrea, and appropriate adjustment of the questionnaire was made before the commencement of the study.

Key informant interviews to fix the remaining information regarding the overall objective of the study and an audit using a checklist to obtain objective data for verification of the corresponding subjective questions in the questionnaire was also done.

### Data analysis

The questionnaires retrieved from the HCWs were screened for completeness, and analyzed using statistical package SPSS Version 22. Statistical analysis such as frequency, mean, and percentages were used, and the data was presented in frequency tables, charts, and graphs. A bivariate analysis was done to test the association between predictor variables and the outcome variable which is NSI using Chi-square while a multivariate analysis using binary logistic regression model was carried out to find out the net effect of the predictor variables to NSI. The level of significance was set at *P* < 0.05 at a confidence interval of 95%.

## Results

Out of a total of 383 healthcare workers (HCWs) who were eligible for the study, 361 HCWs participated in the study. The age of the participants ranged from 21 to 73 years with a mean age of 34.28 ± 12.57 years. The mean work experience of the HCWs was 11.84 ± 12.39 years which range from 1 to 47 years. Higher proportions 64.0% (231/361) of the HCWs were females while more than half (54.6%) of the HCWs were single (Table 1).

**Table 1:** Demographic characteristics of participants (*N* = 361)

Variables		Frequency
**Age group (years)**	≤ 40	259 (71.7%)
	> 40	102 (28.3%)
**Work experience (years)**	1–10	249 (69.0)
	> 10	112 (31.0)
**Gender**	Male	130 (36.0)
	Female	231 (64.0)
**Marital status**	Single	197 (54.6)
	Married	154 (42.7)
	Divorced	10 (2.8)
**Occupational group**	Specialist	22 (6.1)
	General practitioner	30 (8.3)
	Lab workers	26 (7.2)
	Dental workers	20 (5.5)
	Anesthesia nurses	14 (3.9)
	Associate nurses	132 (36.6)
	Registered nurse	97 (26.9)
	Midwives	20 (5.5)

The workplaces of the HCW were Depatment of Medical and Surgical 157 (43.5%), Department of Pediatric 99 (27.4%), Department of Maternity 77 (21.3%) and Laboratory 28 (7.8%) Table 2 [7].

**Table 2:** Workplaces of the participants (*N* = 361)

**Workplace**		Freq. (%)	Total (%)
**Pediatric**	OPD & follow up	15 (4.2)	99(27.5)
	Emergency	19 (5.3)	
	Pediatric ICU	12 (3.3)	
	Neonatology	12 (3.3)	
	Medical Ward	25 (6.9)	
	Surgical Ward	10 (2.8)	
	IOCCA	6 (1.7)	
**Maternity**	OPD and follow up	4 (1.1)	77(21.3)
	Delivery room	19 (5.3)	
	Gynecology Ward	11 (3.0)	
	Obstetrics OR^a^	14 (3.9)	
	Obstetrics Ward	22 (6.1)	
	Gynecology OR^a^	7 (1.9)	
**Medical & Surgical**	Med. OPD & follow up	6 (1.7)	157(43.4)
	Adult Emergency	17 (4.7)	
	Adult ICU	15 (4.2)	
	Med. Ward	24 (6.6)	
	Dialysis Unit	7 (1.9)	
	Surg. OPD & follow up	1 (0.3)	
	Surg. OR^a^ & Recovery	22 (6.1)	
	Surg. Ward	24(6.6)	
	Maxillo-Facial & Dentistry	25 (6.9)	
	ENT OPD & Emergency	6 (1.7)	
	ENT Ward	4 (1.1)	
	ENT OR^a^	6 (1.7)	
**Laboratory**		28 (7.7)	28(7.8)
**Total (%)**		361(100)	361(100)

*OR*^a^ Refers to operation room, *Surg.* Refers to surgical, *Med.* Refers to medical and *ENT* refers to Eye, Nose and Throat, *IOCCA* Refers to international operation center for children in Asmara, *ICU* Refers to Intensive care unit

**Fig. 1:**
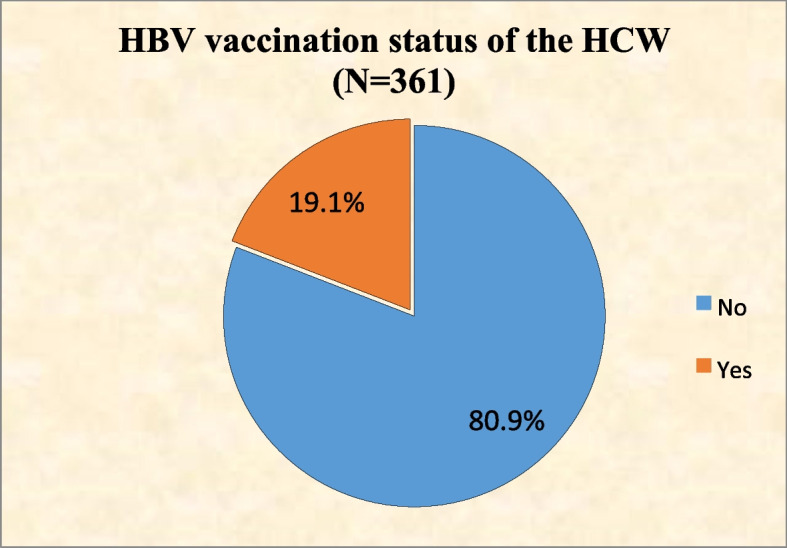
The HBV vaccination status of the HCWs

Figure 1 shows the HBV vaccination status of the HCWs. A total of 69 (19.1%) were vaccinated against HBV.

The respondents were also asked whether they had health problems such as tremor, nervousness, backbone problem, eyesight problem that started in the past 12 months preceding the study (Table 3).

**Table 3:** Health problems among the HCWs (*N* = 361)

Health problems		Frequency (%)
Tremor	No	356 (98.6%)
	Yes	5 (1.4%)
Nervousness	No	350 (97.0%)
	Yes	11 (3.0%)
Backbone problem	No	269 (74.5%)
	Yes	92 (25.5%)
Eyesight problem	No	317 (87.8%)
	Yes	44 (12.2%)

**Fig. 2:**
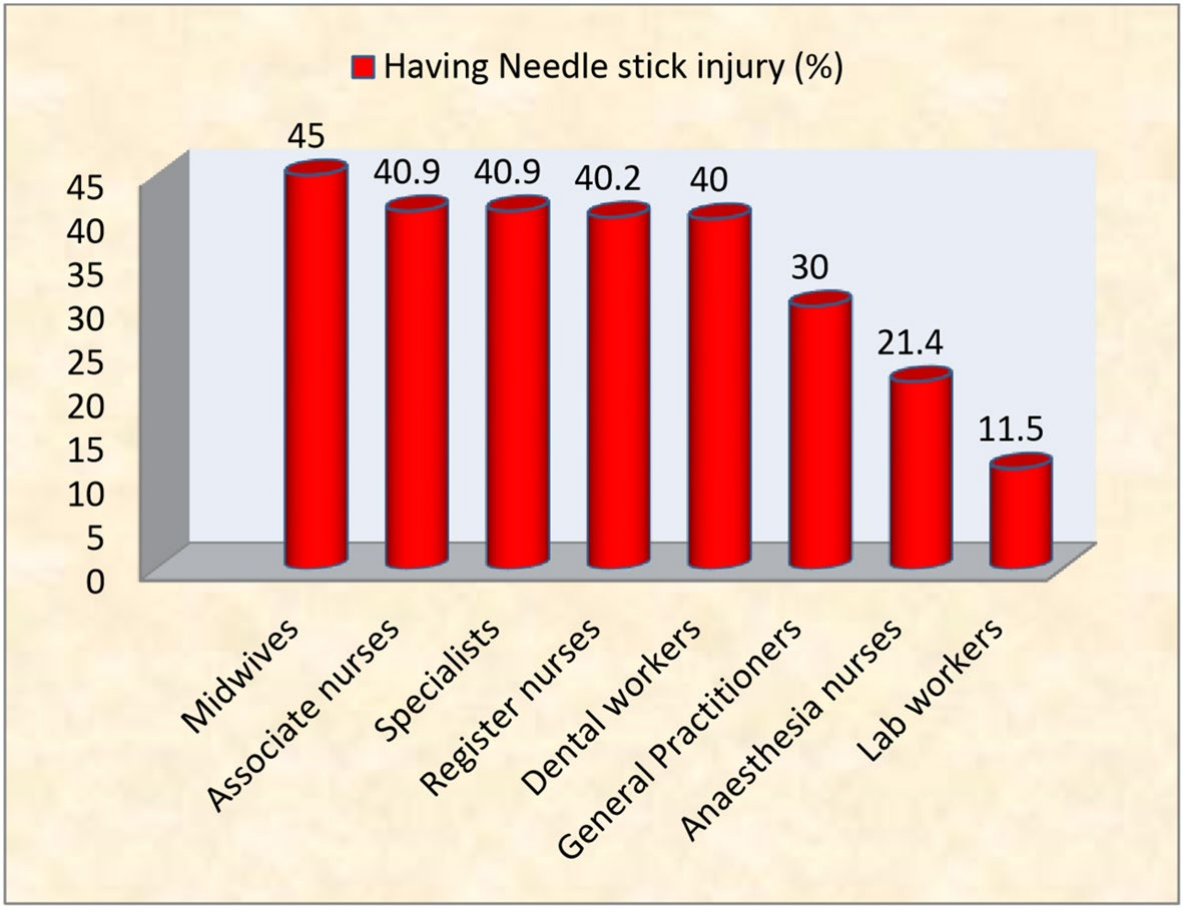
Prevalence of NSI by occupational group (*N* = 134)

Concerning the prevalence of NSI among the different occupational groups, Midwives had the highest prevalence of NSIs (45%). Comparable proportions of NSI were experienced by associate nurses (40.9%), registered nurses (40.2%), specialists (40.9%) and dental workers (40%). However, general practitioners (30%), anesthesia nurses (21.4%) and laboratory workers (11.5%) had lesser occurrence of NSI as compared to the above-mentioned groups (Fig. 2).

Among the different age categories, highest prevalence of NSI was found in the age group ≤ 40 years with prevalence rate of 42.9% as compared to those with age group > 40 years (22.5%) (Table 4).

**Table 4:** Prevalence of NSIs by demographic variables

Variable		Total HCW *N* = 361	HCW who sustained NSI (%)
**Age group**	≤ 40	259	111 (42.9)
	> 40	102	23 (22.5)
**Work experience (years)**	1 to 10	249	106 (43.6)
	> 10	112	28 (24.2)
**Gender**	Female	231	88 (38.1)
	Male	130	46 (35.4)
**Marital status**	Single	197	87 (44.2)
	Married	154	43 (27.9)
	Divorced	10	4 (40)

Prevalence of NSI among the HCW who still practice recapping was 39.4% which was higher than those who don’t recap (33.9%).

The prevalence of NSI in those who reported health problems like tremor (40%), nervousness (45.4%) and backbone problem (51.1%) were higher as compared to their counterparts who did not have such health problems with a prevalence rate of 37.2%, 36.9% and 32.3% respectively (Table 5).

**Table 5:** Prevalence of NSI by work conditions and practices

Variable		Total HCW *N* = 361	HCW who sustained NSI (%)
Working hours per day including the private service	7–8	255	90 (35.3)
	9–10	26	11 (42.3)
	11–12	50	20 (40)
	≥ 13	30	13 (43.4)
Additional duty*	No	239	76 (31.8)
	Yes	122	58 (47.5)
Lack of comfort with work conditions	No	128	54 (42.2)
	Yes	233	80 (34.3)
Frequently of reported stressful work conditions	Always	67	27 (40.3)
	Sometimes	272	100 (36.8)
	Never	22	7 (31.8)
Recapping a needle after use	No	153	52 (33.9)
	Yes	208	82 (39.4)
Vaccine against HBV	No	292	109 (37.3)
	Yes	69	25 (36.2)
Tremor	No	356	132 (37.1)
	Yes	5	2 (40)
Nervousness	No	350	129 (36.9)
	Yes	11	5 (45.4)
Back bone problem	No	269	87 (32.3)
	Yes	92	47 (51.1)
Eyesight problem	No	44	14 (31.8)
	Yes	317	120 (37.8)

Additional duty* refers to any duty like childcaring, private work…etc. that made the HCWs rush during their working hours

Lack of comfort with work conditions* refers to chair, table, light, space …etc.

Majority of the NSI happened in the morning shift with frequency of 78 (58.2%), followed by night shift 32 (23.9%) and afternoon shift 249 (17.9%). The occurrence of NSI varied according to the timing: during procedure constituted the most cause of the NSI with frequency of 81 (60.4%), followed by after use but before disposal, 35 (26.1%) and during disposal, 18 (13.4%) (Table 6).

**Table 6:** Distribution of NSI by work shift, timing and high risk source patient (*N* = 134)

Variable		Frequency (%)
**Work shift**	Morning shift	78 (58.2)
	Afternoon shift	24 (17.9)
	Night shift	32 (23.9)
**Timing**	During procedure	81 (60.4)
	During disposal of sharp	18 (13.4)
	After use but before disposal	35 (26.1)
**High-risk source patient**^a^	Yes	11 (8.2)
	No	89 (66.4)
	Don’t know	34 (25.4)

^a^High-risk patient: patient with history of HIV, HBV or HCV

**Fig. 3:**
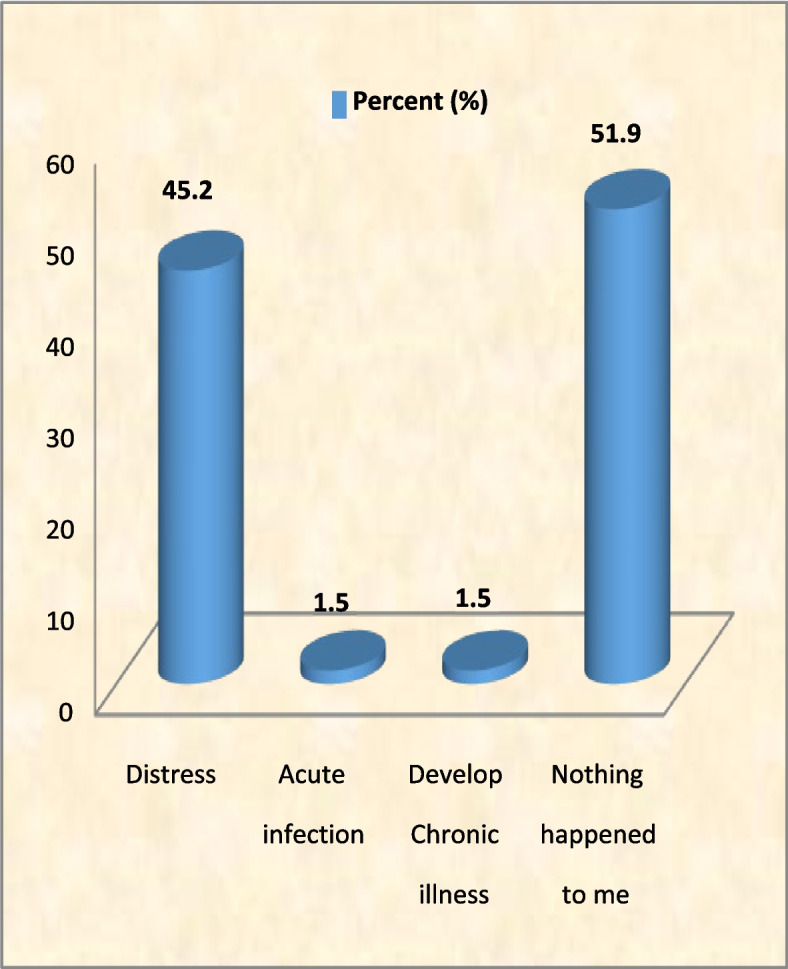
Post exposure (to NSI) health status of the HCW (*N* = 134)

Among the respondents who sustained NSI, different health conditions had been experienced in response to the exposure to NSI where the majority reported distress (Fig. 3).

On chi-square analysis, factors that were found to have significant relationship with NSI include, age (*p* < 0.001), work experience (*p* = 0.001), marital status (*p* = 0.007), additional duties that made the HCW rush during work (*p* = 0.003) and back bone problem (*p* = 0.001) (Table 7).

**Table 7:** Factors associated with NSI among HCW (*N* = 361)

**Variables**		**NSIs**		*p *value
		Yes (%), *N* = 134	No (%), *N* = 227	
Age group	≤ 40	111(42.9)	148 (57.1)	^*^0.001
	> 40	23 (22.5)	79 (77.5)	
Work experience (years)	1 to 10	106 (42.6)	143 (57.4)	^*^0.001
	> 10	28 (25.0)	84 (75.0)	
Gender	Female	88 (38.1)	143 (61.9)	0.609
	Male	46 (35.4)	84 (64.6)	
Marital status	Single	87 (44.2)	110 (55.8)	^*^0.007
	Married	43 (27.9)	111 (72.1)	
	Divorced	4 (40)	6 (60.0)	
Occupational group	Specialist	9 (40.9)	13 (59.1)	0.123
	General	9 (30)	21 (70.0) practitioner	
	Lab worker	3 (11.5)	23 (88.5)	
	D. workers	8 (40)	12 (60.0)	
	Nurses	105 (40)	158(60)	
Working hours per day including the private service	7–8	90 (35.3)	165 (64.7)	0.720
	9–10	11 (42.3)	15 (57.7)	
	11–12	20 (40)	30 (60.0)	
	≥ 13	13 (43.4)	17 (56.7)	
Additional duty	No	76 (31.8)	163 (68.2)	^*^0.003
	Yes	58 (47.5)	64 (52.5)	
Comfort ability with workplace	No	54 (42.2)	74 (57.8)	0.140
	Yes	80 (34.3)	153 (65.7)	
Frequently of reported stressful work conditions	Always	27		0.752
	Sometimes	100 (36.8)	172 (63.2)	
	Never	7 (31.8)	15 (68.2)	
Recapping a needle after use	No	52 (34.0)	101 (66.0)	0.291
	Yes	82 (39.4)	126 (60.6)	
Vaccine against HBV	No	109 (37.3)	183 (62.7)	0.865
	Yes	25 (36.2)	44 (63.8)	
Tremor	No	132 (37.1)	224 (62.9)	0.893
	Yes	2 (40.0)	3 (60.0)	
Nervousness	No	129 (36.9)	221 (63.1)	0.561
	Yes	5 (45.5)	6 (54.5)	
Back bone problem	No	87 (32.3)	182 (67.7)	^*^0.001
	Yes	47 (51.1)	45 (48.9)	
Eyesight problem	No	14 (31.8)	30 (68.2)	0.437
	Yes	120 (37.9)	197 (62.1)	

^*^Refers to significance at .05, *D* workers refers to Dental workers, *An* nurse refers to Anesthesia nurse, *A* nurse refers to Associate nurse and *R* nurse refers to registered nurse

On multivariate logistic regression analysis of the study variables, the factors that were found to be significantly associated with NSI were age > 40, marital status, additional duty that makes HCW rush during their working hours and backbone problem.

Those HCWs in the age group greater than 40 years were 3.2 times less likely to experience NSI than the HCW in the age group 40 years or below. Married HCW were 1.7 times less likely to experience NSI than unmarried HCW. HCW who had additional duty that makes them rush during their working hours were 2.1 times more likely to get NSI HCWs who did not have additional duty. Similarly, HCW who had back bone problem been 2.2 times more likely to get NSI (Table 8).

**Table 8:** Multivariate logistic regression analysis of factors associated with NSI among the HCW

**Variables**		Adjusted odds ratio (AOR)	
		AOR	*p-*value
Age group	≤ 40	1	
	> 40	0.314	0.050
Marital status	Single	1	
	Married	0.595	0.050
	Divorced	1.365	0.680
Additional duty	No	1	
	Yes	2.159	0.002
Back bone problem	No	1	
	Yes	2.323	0.001

OR = 1 is the reference category

### Response to exposures

**Fig. 4:**
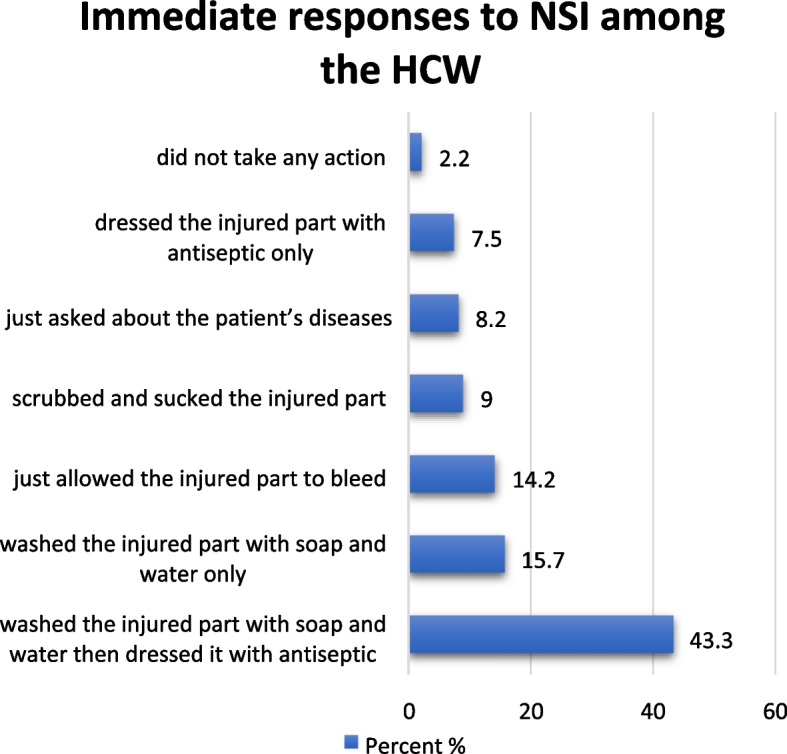
Immediate responses of the HCW right after sustaining NSI (*N* = 134)

As an immediate response to NSI, 43.3% of the HCW washed the injured part with soap and water and dressed it with antiseptic while only 2.2% preferred to ignore the injury (Fig. 4).

Among the occupational groups, Specialists (33.3%), General Practitioners (33.3%), Anesthesia nurses (33.3%), and Midwives (33.3%) were less likely to report NSI as compared to Dental workers (37.5%), Registered nurse (41.1%), and Associate nurses (44.4%)(Table 9).

**Table 9:** Proportion of reported NSI by occupational group

Occupational group	Having NSI	Reported NSI (%)
Specialists	9	3 (33.3%)
General practitioners	9	3 (33.3%)
Anesthesia nurses	3	1 (33.3%)
Midwives	9	3 (33.3%)
Associate nurses	54	24 (44.4%)
Registered nurses	39	16 (41.1%)
Dental workers	8	3 (37.5%)

**Fig. 5:**
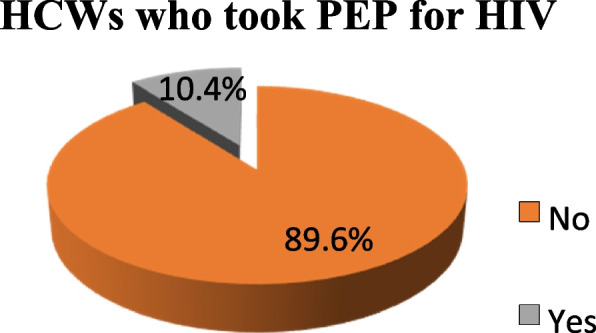
HCWs who took PEP for HIV after NSI (*N* = 134)

Out of the 134 respondents who suffered NSI, only 14 (10.4%) took HIV post exposure prophylaxis (Fig. 5). Concerning the occupational categories of the HCW who took HIV post exposure prophylaxis, 7 (50%) were Associate nurses, 5 (35.7%) Registered nurses and the remaining 2 (14.3%) Specialist Doctor.

### Key informant interviews and audit results of checklist

In addition to the questionnaire used as data collection tool, checklists and key informant interviews were employed to complete the remaining information regarding the overall objective of the study. Some of the information obtained through them included the following:

Occupational exposure control activities are partly undertaken by infection control committees in ONRH. Trainings on infection prevention and injection safety have been held although few and irregular. Among the reasons stated for not conducting such training routinely at regular basis were: high workload and understaffing which probably kept the available HCW too busy not to participate in the training. In addition, according to discussions with the key informants, mainly matrons, there was low motivation in the HCW to participate in infection prevention and injection safety trainings. This was because of lack of reward to encourage the HCWs.

**Fig. 6:**
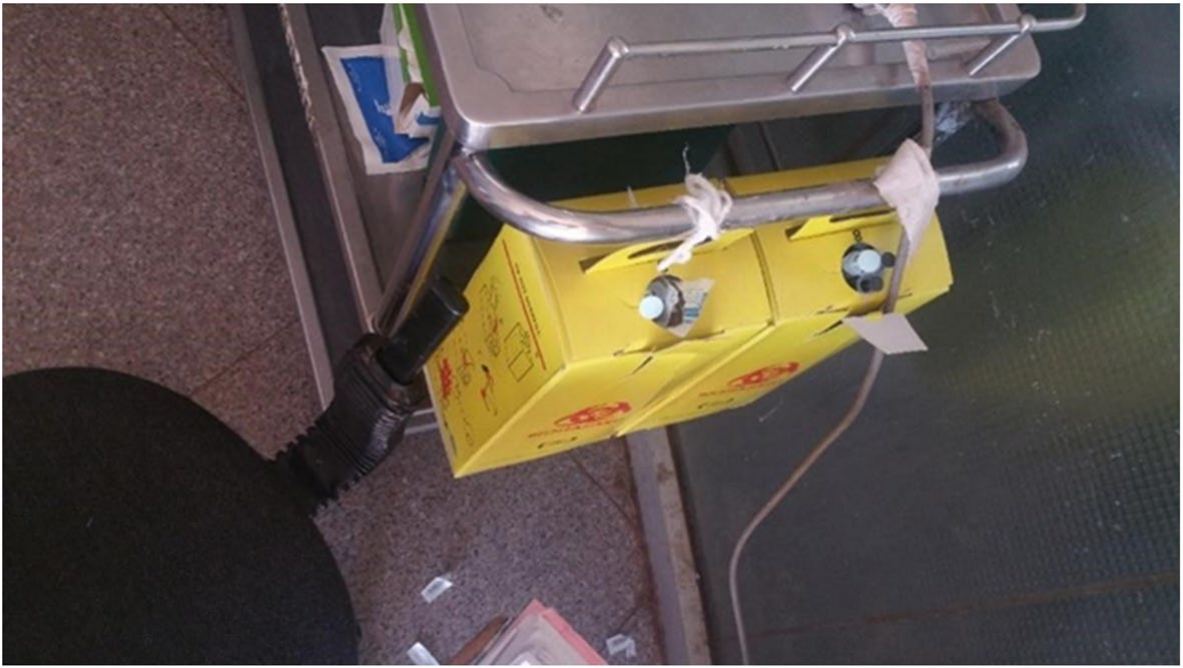
Filled sharp box in the adult emergency of the hospital

During visits by the researchers at the hospital departments, personal protective equipment was available, except for boots in the Gynecology and Obstetrics ORs. Sharps containers (Fig. 6.) were also available, however, they were not puncture proof (but disposable) and the challenge of leaving them until filled (overflowing) was observed in Pediatric Surgical Ward, Pediatric OPD and follow up clinic, Pediatric emergency, Adult emergency, Adult medical ward, Gynecology OR, Obstetric OR and Delivery room.

## Discussions

In this study, it was found out that 37.1% (134 out of 361) of HCW had sustained NSI at some time in the last 12 months preceding the study, implying that HCW in the study hospitals were at risk of contracting blood-borne diseases due to NSI. This 12-month prevalence in this study was much lower than the studies done in Egypt [16], Congo [17] and India [18] where prevalence rate were 67.9%, 45% and 80.1% respectively. However, it was found to be higher when compared to studies conducted in Ethiopia [19], Switzerland [20], Malaysia [21] and South Africa [22] where the 12 months prevalence of NSI was 31%, 9.7%, 23.5%, and 23.5% respectively. The high prevalence of NSI in the study could be because there were not enough interventional efforts in infection prevention and safety activities.

Although all HCWs in contact with sharp medical devices were at risk of exposure to blood and other body fluids, there was high prevalence of NSI in midwives where 45% of them have experienced it in the preceding one year. This may be attributed to the discomfort experienced in their workplace where 75% of them, which was the highest figure among the respondents, reported that they were not at ease with delivery coaches and light. Such discomfort was verified through the checklist. In addition, it was found out that working for more hours per day was significantly related with NSI among the Midwives (*p* = 0.04) which was not true in the other HCWs. Hence, possible explanation could be that the high report of discomfort in the workplace may add up with the long working hours to result in fatigue, loss of concentration and thereby high prevalence of NSI as compared to other HCWs. Additional possible explanation for the high cases of NSI in midwives, as obtained from the matron, could be the non introduction of safer alternatives to sharp tipped suture needles.

In this study, the mean number of NSI per HCW in the past 12 months was 1.89 (± 1.04 SD) and this is lower when compared with a study in India where mean number of NSI was as high as 4.5 (± 3.4 SD) [23]. A large multinational study by WHO on global burden of sharps injury estimated the average number of injuries per HCW to be 0.2–4.7 sharps injuries per year [7].

In this study, the prevalence of NSI was found to decline with increase in age. HCW in the age group < 40 years had higher prevalence of NSI (42.9%) as compared to those with age group > 40 years (22.5%). Age greater than 40 years was significantly associated with NSI (AOR = 0.314, *p* = 0.05). This is comparable to a study conducted in Egypt that showed NSI being reduced with increasing age [16]. Another study conducted in Turkey in 2008 also reported that young age was a risk factor for occupational injuries [24]. This is possibly due to limited professional experience and the fact that young HCW tend to show negligence in their work which led to increased risk of NSI.

This study showed that the prevalence of NSI was high among the HCW with work experience of 1 to 10 years (43.6%) as compared to those above 10 years (24.2%). And this was statistically significant (*p* = 0.001). This may be attributed to the fact that more HCW with less than 10 years of work experience reported that they still practiced needle recapping (43.6%) and had additional duties that made them rush during working hours (34.8%). A study conducted among nurses in 2002 in USA showed that the probability of ever having a NSI was inversely related to years of experience [25]. This fact was endorsed in the present study too.

In the current study, NSIs were more frequently reported by females (Prevalence of 38.1%) as compared to males (35.4%). Despite the absence of a statistically significant relationship between gender and NSI, similar results have been previously reported [26]. For example, in a study conducted in two tertiary hospitals of Pakistan, high prevalence of NSIs were reported by female HCW (67.1%) as compared to their male counterparts (57.3%) [27]. In contrast to this result, another study conducted in Gojjam, Ethiopia showed that male workers were more victims as compared with female workers with a prevalence of 27.5% and 11.3% respectively [28]. The reason they stated for such difference was ‘may be disparities in socioeconomic development of the HCW’ which was computed as associated factor in their study.

As this study showed, HCWs who were unmarried during the study had higher prevalence of NSI (44.2%) as compared to the married (27.9%) and divorced (40%). A similar study carried out in Ethiopia also revealed that unmarried HCW had higher prevalence of NSI (30.5%) than married (15%) and divorced (4.8%) [28]. In the present study, marital status was statistically associated to NSI (AOR = 0.595, *p* = 0.05).

Respondents working in the adult ICU were found to have higher prevalence of NSI (61.5%) as compared to other departments in the hospital. One possible explanation as obtained from the checklist was that there was reduced light which probably predisposes the HCW to NSI. Other possible explanations given by head nurses could be due to stressful work conditions as critical patients were admitted there. Similar results were obtained in the United States of America [29] where it was observed that the proportion of injuries in intensive care units from suture needles rose significantly.

In this study, majority of exposures occurred during the morning shift (58.2%). This may be attributed to the busy schedule (high patient flow) at the time and the pressure among staff to complete tasks. In addition, majority of the activities such as diagnosis, laboratory tests, operations and treatments are performed in the morning shift. Similar study done in India analysis of 411 recorded exposures demonstrated that more people were exposed between 9.00 am and 11.00 am [30]. another study showed that only 25% of the NSI occurred in the morning shift which is lower as compared with the figure in the present study [31].

Among the procedures that placed HCWs at risk of NSI, suturing was the highest (30.9%), followed by IV injection (29.6%). This was consistent with the findings of same study in Rift Valley Provincial Hospitals, Kenya where suturing was the highest (29%) [32]. A similar study done in Germany also showed that suturing caused most of the NSI (23%), followed by I.V injection (13%) [33]. On the other hand, studies conducted in India [30] and Pakistan [34] showed that blood withdrawal caused 55% and 41.2% of the NSI, respectively.

The United States national surveillance system for health care workers identified six devices that were responsible for the majority of NSI, these were syringe needle (32%), suture needle (19%), winged steel needle (12%), scalpel blades, intravenous catheter stylets and phlebotomy needles (3%) [35]. This study also revealed that syringe needle was a major cause of the NSI (61.9%). This is much higher as compared to the study done in Alexandria hospitals, Egypt (38.4%) [16]. However, it is also much lower when compared with a study conducted in War Memorial hospital, Kenya where the comparative figure was 80% [32]. Another study conducted in India showed that 62% of exposures to blood and body fluids involved syringe needle [36]. This implies that injuries with syringe needle might be due to inappropriate needle handling. It might also be due to the fact that majority of the procedures done to patients require syringe needles. And this probably reduces the attention during injection and may put HCW under higher risk of injuries. According to the WHO, about 90% of the medical syringes are used to administer drugs, 5% for vaccinations and 5% for other uses such as blood transfusions [37].

In this study, the most important situations that gave rise to NSI and which the respondents thought so were handling uncooperative patients (20.9%) and recapping of needles (19.4%). Similar study carried out in India showed that 66.3% of the HCW received the NSI due to recapping needles and 13% when handling uncooperative patients [30]. This result was in agreement with another similar study done in a secondary care hospital in Saudi Arabia where recapping caused most of the NSI (29%), followed by collision with sharps (14%) and disposal related (11%) [38]. Another similar study conducted in Nigeria also showed that recapping of needles (38.0%) and patient aggression (26.0%) were the most common circumstances leading to NSI in Accident and Emergency departments [39].

In the current study, 8.2% of the HCWs had sustained NSI from high-risk source patients (those who had history of infection with HIV, HBV or HCV). This is similar to a study carried out in Egypt where 8.2% of the NSIs came from high-risk source patients [16].

Despite instructions given to HCW not to recap the needles, it was still a common practice, as 42.4% of the participants were recapping. This result was found to be lower than the findings of a study done in Uganda [40] and Ethiopia [19] where 50% and 74.7% of the HCW were recapping most or all the time respectively. In this study, needle recapping was found as the second most common cause that resulted in 19.4% of NSI. This compares favorably with a study in Kenya which found out that recapping of needles was the second leading cause of needlestick injuries, which caused 20.2% (19/94) of the total injuries by medical sharps [41]. Several studies have shown recapping to be an important cause of NSI [18, 42, 43]. Recapping of needles has been prohibited under the Occupational Safety and Health Administration (OSHA) blood-borne pathogen standard [44].

A frequent argument against safer devices is the higher price compared with conventional sharps. Nevertheless, besides the commercial relevance there are ethical values such as protecting the health of hospital staff from known risks and harm that should not be ignored [45].

This study showed that only 15.7% of the HCW washed the injured part with soap and water, which is the right response measure. This is probably due to lack of knowledge about what immediate action to take. Similar study conducted in Hyderabad, India showed that 66% of the HCW who sustained NSI said that they had washed the injured part with soap and water, while 47% applied spirit/alcohol [30].

This study showed that 59% of the HCW did not report the NSI. This is consistent with the report that 40–70% cases of NSI are unreported in developing countries [5]. Similar studies done in Ethiopia [19] and Germany [33] revealed that 53.9% and 50.4% of the HCW, respectively, didn’t report their injury to concerned bodies. Unreported NSIs are a serious problem and prevent injured HCWs from receiving PEP against HIV, which is shown to be 80% effective against HIV infection [46].

In the present study, the most common reasons stated for not reporting included: *I just prefer to take care of it myself* (32.1%), *Believe that I was at a lower risk of infection* (23.1%) and *No need to report* (21.8%). This is higher in comparison to studies done in Ethiopia [19] and Egypt [16] where those who said, *I believe that I was at a lower risk of infection,* were 12.6% and 19.9%, respectively. However, it was a risky perception because a person who looks healthy doesn’t necessarily mean he/she is free from communicable diseases such as HIV, Hepatitis B and C. Since every patient/client should be considered and treated as infectious.

In this study, 1.5% of the HCW reported that they were infected with chronic diseases because of NSI. However, none of them specified the disease he/she acquired. This may be due to stigma associated with diseases such as HIV/AIDS. It is estimated that about 4.4% (0.8%–18.5%) of HIV infections among HCW may be attributable to occupational sharps injuries worldwide [47].

Among the 134 HCW who suffered NSI, only 10.4% took HIV post exposure prophylaxis. This figure is much lower as compared to that reported in a study conducted in Rift Valley Provincial Hospitals, Kenya where 25% of the injured HCW took PEP [32]. However, it is higher when compared to that reported in a study carried out in India where only 7.8% of HCW took a course of PEP [23]. As most HCW did not report the exposures, they were not evaluated for indication of PEP, therefore it is important to note that the number required to take PEP may not be exact.

This study indicated that only 19.1% of the respondents had been vaccinated against hepatitis B virus (HBV) previous to this survey. This finding was much lower when compared with the studies conducted in Germany [33], Pakistan [48], Saudi Arabia [49], India [50] and Egypt [51] that showed the vaccination coverage rates for hepatitis B were 78.2%, 45%, 84%, 82% and 87.1%, respectively. This may reflect poor accessibility and affordability of HBV vaccine in our country. According to the WHO estimates, vaccination coverage varies from 18% in Africa to 77% in Australia and New Zealand [52]. However, considering the high risk of hepatitis B and C in HCW compared to general population, this poor vaccine coverage puts HCW under increased risk of infection and warrants immediate attention by policy makers.

According to this study, NSIs are still common and a concern among HCWs. The National Institute of Occupational Safety and Health, United States, identified the following as predisposing factors to needle stick injuries: over-use of injections and unnecessary sharps, lack of supplies (disposable syringes, safer needle devices, sharps disposal containers), lack of access and failure to use sharps container immediately after use, poorly trained staff, needle recapping, no engineering control, such as safer needle devices, passing instruments from hand to hand as in an operating room, and lack of hazard awareness and training [53]. This is in agreement with findings from this study in which 42.4% of the HCW recap a used needle most or all the time, improper disposal of the sharps, lack of training on infection control and safety, lack of supplies such as safer needle devices, and lack of facilities like light.

It is estimated that sharp injuries could be reduced by 70% if recapping was avoided and needles were disposed promptly into puncture resistant containers [54].

## Conclusion

The study concluded that there is high occurrence of NSIs among healthcare workers where over one third of the respondents experienced NSIs. This implies that about one out of three HCW sustained NSI per year. It was also revealed that midwives had the highest prevalence of NSI as compared to the other health professionals.

The factors that were found to be predictors of NSI at the hospital include age less than 40 years, being unmarried, additional duty that made the HCWs rush during their working hours and backbone problem.

Only few HCWs took the right response measures right after they sustained the NSI.

The hospital has partly adopted the use of personal protective equipment and provision of post exposure prophylaxis. However, there is no safety engineered devices, HBV vaccine, frequent and regular training on infection prevention and safety available in the hospital. There is also low reporting of NSI where more than half of the respondents who sustained the injury didn’t report. Besides there was low utilization of PEP and noncompliance with standard disposal of sharps among the HCW. Discomfort in work station designs such as chair, table, delivery coach, and space were evident in some work sites of the hospital.

## Recommendations

Several recommendations to be implemented at the hospital were identified as discussed below.

Special attention should be given to the midwives, specialists, associate nurse, registered nurse, dental workers.

- Unmarried HCW, those in the age < 40 years, those HCW with backbone problem, those who have additional duty that makes them rush during their working hours needs serious supervision,
- The infection control committee should ensure that all HCW are trained, sensitized, and updated on issues related to NSI risk reduction.
- Hepatitis B vaccination is recommended for HCW, and the hospital should provide mandatory immunization program for their HCW.
- Enhancing workers safety by providing safety devices such as auto disable/retractable needles and blunt sutures. Workplace designs such as chair, table, delivery coach, space should also be designed in a way that suits the HCW.
- The infection control unit should ensure that those who are injured and require post-exposure prophylaxis especially during night shift get the PEP in time (within 2 h of exposure is most effective).
- Since NSI are often underreported, health care institutions should not underestimate it.

Further research is needed to assess on the following areas:

- The extent of needlestick injury among housekeeping staffs such as cleaners, laundry workers and waste handlers at the hospital.
- NSIs Health effects on HCWs, by following up of participants.

## Limitations

This study was conducted in only one hospital of Eritrea. It would have been more desirable if it had been conducted in different hospitals of the country to gain a fair idea of the different factors that expose HCWs to NSI. Moreover, the study was done within two months and therefore there was no sufficient time to do follow up of HCWs health impacts after their injury. We believe, however, that our findings could add to the body of knowledge on the subject.

### Supplementary Information

Supplementary Material 1.

## Data Availability

The complete data set supporting the conclusion of this article is available from the corresponding author and can be accessed upon reasonable request.
