# Supplementary material for: Prevalence, response and associated factors of needlestick injury among health care workers in Orotta National Referral Hospital, Eritrea
Source: BMC Health Serv Res. 2024 Jul 27;24:853. doi: 10.1186/s12913-024-11255-x (PMC11282850; doi:10.1186/s12913-024-11255-x)
Supplement: Supplementary file 1 — Supplementary Material 1. [file 12913_2024_11255_MOESM1_ESM.docx]

**Appendices**

**Appendix 1: Questionnaire**

Date ___/___/2017

Questionnaire about “Prevalence, response and associated factors of sharp injury among health care workers in Orotta National Referral Hospital”

Anonymous Self-Administered Questionnaire No: ___________

**Introduction:**

First and foremost, we are thankful for your attention and patience to hear our request for your participation in our study. This is a research work on the prevalence of **sharp injury** among **health care workers** (HCW) in Orrota National Referral Hospital (ONRH) which is done by students of Public Health as partial fulfillment of the requirements for a B.Sc. in the School of Public Health. The findings of this study is expected to be helpful to HCW, the Ministry of Health and other concerned bodies as a base to come up with necessary programs and policies to amend the gaps that this study will identify and thereby make the jobs of the concerned bodies easier in following the appropriate measures and effective interventions in minimizing the existence of sharp injury. We are kindly requesting your consent to participate in this study. Your privacy as well as confidentiality will be assured. Please make a ‘tick’ on either of the boxes provided below to make us know your decision.

**Yes I agree to participate No I don’t agree to participate**

Those who are willing to participate you can proceed to the following questions and instructions. We are hopefully expecting you to be honest in your answers so that the result of this study will be representative and profitable for the concerned ones including HCW, patients, MOH etc...

**Part I: Personal Particulars**

1. Age: __________

2. Years of work experience: ___________

3. Gender: 1. Male 2. Female

4. Marital status: 1. Single 2. Married 3. Divorced 4. Widowed

5. Occupational group:

1. Specialists 4. Registered nurses 7. Dental workers

2. General practitioners 5. Associate nurses 8. Lab workers

3. Anesthesia nurse 6. Midwives

6. Workplace:

**1. Pediatrics:** a. OPD and follow up clinic b. Emergency c. Pediatric ICU

d. Neonatology e. Medical Ward f. Surgical

g. IOCCA

**2. Obstetrics & Gynecology:**

a. OPD and follow up clinic b. Delivery room

c. Gynecology Ward d. Obstetrics Ward e. Gynecology

**3. Internal medicine:** a. OPD and follow up clinic b. Emergency c. ICU

d. Ward e. Dialysis Unit

**4. Surgical:** a. OPD and follow up clinic b. Emergency c. ICU

d. OR e. Surgical

f. Maxillo Facial & Dentistry

**5. ENT:** a. **OPD** & Emergency b. Ward c. OR

**6. Laboratory**

**Part II: Work Conditions and Practices**

1. Do you use sharp objects in your current work in the hospital?

1. Yes 2. No

**If your answer to # 1 above is “Yes”, pass to # 2 below but if it is “No”, stop here (Leave the questionnaire).**

2. How frequently do you wear glove while working with sharp objects?

1. Always 2. Sometimes 3. Never

3. If you wear glove, how many gloves do you wear on one hand?

1. Single 2. Double 3. Triple

4. How many hours do you work in a day including the same task you do in a private service?

1. 7-8hrs 2. 9-10hrs 3. 11-12hrs 4. More than 13 hrs

5. Do you have additional duty **which makes you rush** in your working time (e.g. child caring, overtime work opportunity etc...)?

1. Yes 2. No

6. Are you given periodic/regular training to improve your skills in safe systems of work related to sharps in the hospital?

1. Yes 2. No

7. Are you comfortable with your chair/table/work place design such as lighting and space during your working hours?

1. Yes 2. No

**If your answer is ‘No’, specify the typical discomforts__________________________________________**

8. How frequently do you get stressful / pressurized work conditions?

1. Always 2. Sometimes 3. Never

9. Do you recap used needle?

1. Yes 2. No

10. Have you ever received hepatitis B virus (HBV) vaccine in your hospital or any other place?

1. Yes 3. No

11. Which of the following health problems that begun in the last 12 months do you have?

A. Tremor E. Eye sight problem I. No health problem

B. Insomnia F. Stress

C. Nervousness G. Hearing problem

D. Back bone problems H. Others______________________________________________

**N.B. In this questionnaire we mean by sharp injury as a skin injury in which hollow bore needle or sharp instruments, including but not limited to needles, lancets, scalpels, and other sharp objects that are contaminated with body fluids, accidentally penetrates the skin during your work time.**

**Please answer the following questions if and only your choice to # 1 is ‘yes’.**

**When you had more than one sharp injury in the last 12 months, please consider the most recent one (the latest injury you had experienced) when answering the following questions below:**

**Part III: Sharp Injury Events**

1. Have you ever been injured by sharps when working **within the last 12 months only**?

1. Yes 2. No

2 How many times did you get sharp injury when working in the last 12 months only?

1. Once 2. Twice 3. Three times 4. More than three times

3 What kind of sharp object had you been injured with? 1. Scalpel 3. Syringe needle 5. Suture needle 2. Winged, butterfly needle 4. Lancet 6. Stylets of IV cannula. **If other, specify__________________________________________**

4 What was the work shift during which the injury happened? 1. Morning shift 2. Afternoon shift 3. Night shift

5 When did the injury happen?

1. During procedure 3. After use but before disposal

2. During disposal of the sharp

6 **If only** your answer to # 5 above is ‘**during procedure**’ then what procedure were you performing?

1. Blood withdrawal 4. Surgery 7. Sewing/suturing

2. I.V injection 5. I.M injection 8. S.C. injection

**If other procedure, specify________________________________________________________________**

7 What was the cause of the injury?

1. Recapping of needle. 6. Handling uncooperative patient.

2. Collision with another health worker. 7. Passing equipment to another health worker.

3. Collection and disposal of sharp wastes. 8. Collision with sharp object.

4. Working with reduced lighting.

**If other, specify________________________________________________________________________**

8 Was the sharp from a high-risk patient (history of infection with HIV, hepatitis B virus [HBV] or hepatitis C virus [HCV] or injecting drug user)?

1. Yes 2. No 3. Don’t know

**If ‘Yes’ specify as (HIV, HBV. HCV etc...) ____________________________________________________**

9 Where did the injury occur?

**1. Pediatrics:** a. OPD and follow up clinic b. Emergency c. Pediatric ICU

d. Neonatology e. Medical Ward f. Surgical

g. IOCCA OR

**2. Obstetrics & Gynecology:** a. OPD and follow up clinic b. Delivery room

c. Gynecology Ward d. Obstetrics OR

e. Obstetrics Ward f. Gynecology OR

**3. Internal medicine:** a. OPD and follow up clinic b. Emergency c. ICU

d. Ward e. Dialysis Unit

**4. Surgical:** a. OPD and follow up clinic b. Emergency c. ICU

d. OR e. Surgical

f. Maxillo Facial & Dentistry

**5. ENT:** a. OPD & Emergency b. Ward c. OR

**6. Laboratory**

**If other, specify_______________________________________________________________________________**

10 What happened to you after you get the injury? More than one answer is possible.

A. Distress C. Develop Chronic illness

B. Acute infection D. Nothing

**Specify the diseases or infection if you get____________________________________________________**

11 Did you report to any responsible body?

1. Yes 2. No

**If your answer is ‘yes’, please specify to whom you reported:____________________________________**

12 If your answer is **‘No’** then what was the main reason for not reporting? **Encircle one main reason**.

1. Fear of punishment by employer. 6. Shortage of time.

2. No access to HIV post-exposure prophylaxis. 7. No reporting system.

3. Belief that I was at low risk of infection. 8. Did not know whom to report.

4. I just prefer to take care of it myself. 9. No need to report.

5. Belief that my hepatitis B vaccination status was sufficient.

**If other, specify________________________________________________________________________**

13 What response measures did you take immediately after injury? Please encircle **only one** of the following choices.

1. I washed the injured part with soap & water only.

2. After I washed the injured part with soap & water, I dressed it with antiseptic.

3. I just dressed the injured part with antiseptic only.

4. I scrubbed and sucked the injured part.

5. I asked about the patient’s disease.

6. I just allowed the injured part to bleed.

7. I did not take any action.

**If other, specify________________________________________________________________________**

14 Did you take a post exposure prophylaxis for HIV after the injury?

1. Yes 2. No

Thank you!
